# Non-Local Means Denoising of Dynamic PET Images

**DOI:** 10.1371/journal.pone.0081390

**Published:** 2013-12-05

**Authors:** Joyita Dutta, Richard M. Leahy, Quanzheng Li

**Affiliations:** 1 Center for Advanced Medical Imaging Sciences, Department of Radiology, Massachusetts General Hospital, Boston, Massachusetts, United States of America; 2 Signal and Image Processing Institute, Department of Electrical Engineering - Systems, University of Southern California, Los Angeles, California, United States of America; University of Navarra, Spain

## Abstract

**Objective:**

Dynamic positron emission tomography (PET), which reveals information about both the spatial distribution and temporal kinetics of a radiotracer, enables quantitative interpretation of PET data. Model-based interpretation of dynamic PET images by means of parametric fitting, however, is often a challenging task due to high levels of noise, thus necessitating a denoising step. The objective of this paper is to develop and characterize a denoising framework for dynamic PET based on non-local means (NLM).

**Theory:**

NLM denoising computes weighted averages of voxel intensities assigning larger weights to voxels that are similar to a given voxel in terms of their local neighborhoods or patches. We introduce three key modifications to tailor the original NLM framework to dynamic PET. Firstly, we derive similarities from less noisy later time points in a typical PET acquisition to denoise the entire time series. Secondly, we use spatiotemporal patches for robust similarity computation. Finally, we use a spatially varying smoothing parameter based on a local variance approximation over each spatiotemporal patch.

**Methods:**

To assess the performance of our denoising technique, we performed a realistic simulation on a dynamic digital phantom based on the Digimouse atlas. For experimental validation, we denoised 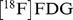 PET images from a mouse study and a hepatocellular carcinoma patient study. We compared the performance of NLM denoising with four other denoising approaches – Gaussian filtering, PCA, HYPR, and conventional NLM based on spatial patches.

**Results:**

The simulation study revealed significant improvement in bias-variance performance achieved using our NLM technique relative to all the other methods. The experimental data analysis revealed that our technique leads to clear improvement in contrast-to-noise ratio in Patlak parametric images generated from denoised preclinical and clinical dynamic images, indicating its ability to preserve image contrast and high intensity details while lowering the background noise variance.

## Introduction

Positron emission tomography (PET) is a 3D imaging technique that uses targeted radioisotope-labeled tracers to visualize vital physiological information, such as metabolism, blood flow, and neuroreceptor concentration [1]–[3]. Quantitative interpretation of PET images is crucial both in diagnostic and therapeutic contexts. Dynamic PET imaging, which reveals information about radiotracer kinetics in addition to the spatial distribution, is immensely promising for PET quantitation. Unlike static PET, where the coincidence events are summed up over the entire scan duration and a 3D spatial image is generated, dynamic PET yields a 4D spatiotemporal map of tracer distribution by binning these events over multiple time frames of shorter durations. A dynamic frame-by-frame reconstructed PET image thus consists of a set of voxel-wise time activity curves (TACs), each of which represents the time course of tracer activity corresponding to a voxel location in the image. The predominant methodology for quantitative interpretation of dynamic PET images involves the fitting of compartmental models [4], [5] to the TACs. This approach uses a set of coupled differential equations to describe tracer exchange between different compartments (physically or chemically distinct states of the tracer). The end result is a spatial map of either kinetic microparameters, which are the rate constants associated with inter-compartmental tracer exchange [6], [7] or, alternatively, of some macroparameters, which are physiologically meaningful functions of the underlying microparameters [8], [9]. Images of kinetic micro- or macroparameters are quantitative and particularly promising for a wide range of applications, including longitudinal studies and cancer management [10], [11], cardiac and cerebral perfusion studies [12], [13], and pharmacokinetic and pharmacodynamic studies [14].

In order to track the rapid change in tracer activity immediately after its administration, dynamic frame-by-frame PET imaging tends to use shorter time bins in earlier parts of a scan. As a result, compared to static PET images where the emission events are binned over relatively long periods of time, dynamic PET images corresponding to earlier time frames have fewer photon counts per frame and tend to be substantially noisier. In applications where a region-of-interest (ROI) can be clearly identified, TACs corresponding to all the voxels within the ROI are averaged before parametric fitting leading to significant reduction in noise. In comparison, voxel-by-voxel estimation of parametric images is more susceptible to the effect of noise. This necessitates a denoising step prior to kinetic analysis of dynamic PET images. In this paper, we present a denoising technique based on *non-local means* (NLM) which, as we demonstrate, allows us to denoise reconstructed TACs without causing significant increase in bias.

Our work was inspired by the highly-cited seminal paper by the Buades et al. [15] which proposed the NLM denoising approach and evaluated its performance against an array of existing approaches (including Gaussian, anisotropic, total variation, wavelet, and several other types of filtering) based on a number of metrics, with a special emphasis on method noise, defined as the difference between a digital image and its denoised version. Since then, a wide spectrum of papers on this topic have appeared, some proposing algorithmic modifications [16]–[20] and some others extending the underlying theory [21]–[24]. The technique has also been extended to video denoising applications [25], [26] and to non-Gaussian noise removal [27]–[30]. The NLM technique exploits self-similarities in images by comparing local neighborhoods. The similarity between a given voxel pair is robustly derived from intensity differences between the patches of neighboring voxels surrounding them. Mathematically, the NLM filter has been viewed as a diffusion or a graph Laplacian operation in patch intensity space [22], [31] and as a Bayesian estimator [21]. Its appeal lies in its superior performance in spite of its simplicity compared to more sophisticated global and multiscale denoising approaches. In the medical imaging community, the technique has been used for MR image denoising [16], [32], [33], CT image denoising [34], SPECT image denoising [35], PET image denoising by incorporating anatomical information [36], and priors for PET image reconstruction [37], [38]. In this paper, we present an NLM filter for dynamic PET imaging, an arena where the need for denoising is particularly pressing. We offered a proof of concept for our idea in our earlier paper on this topic [39] in which temporal patches were used for similarity computation for NLM. In this work, we extend this idea to include spatiotemporal image patches thereby establishing a more robust denoising approach.

The overall objective of this work is to develop an NLM-based denoising framework for dynamic PET images and to assess the quantitative and qualitative merits of the resultant approach relative to other well-known image denoising approaches. We compare this technique with Gaussian denoising and principal component analysis (PCA) based denoising, both widely used in the context of dynamic PET imaging. In addition, we compare this technique with HighlY constrained backPRojection (HYPR) and conventional NLM denoising based on spatial patches. We perform a realistic simulation study based on a dynamic digital mouse phantom and compare the denoising methods by examining bias-variance characteristics of the denoised dynamic images and the corresponding Patlak parametric images. We then apply the developed method to denoise a preclinical 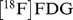 PET dataset from a mouse study and a clinical 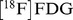 PET dataset from a patient with hepatocellular carcinoma and perform Patlak analysis on these datasets.

## Theory

### The NLM Filter

The NLM filter formulated by Buades et al. restores a given pixel by computing a weighted average over pixel intensities with the weights determined by a robust similarity metric derived from the local neighborhoods of the pixels. Mathematically, the NLM-denoised intensity, 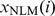, corresponding to the 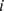
th pixel, is given by the weighted average:
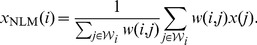
(1)


Here 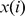 represents the original intensity of the 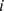
th pixel and 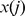 represents the intensity of any pixel lying within a rectangular search window, 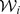, of a fixed size centered at the 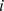
th pixel. The weight, 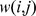, is a measure of the similarity between the immediate local neighborhoods surrounding the 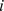
th and 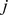
th pixels. These local neighborhoods, referred to as *patches* by Buades et al., are square windows (smaller than the search window 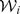) around a given pixel, usually of size 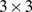, 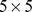, or 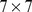. The underlying assumption here is that similar patches have similar central pixels. The squared 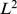 norm of the intensity differences between the vectorized patches for a pair of pixels is converted to a similarity measure using an isotropic Gaussian kernel as follows:
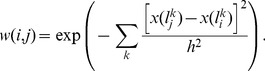
(2)


Here, 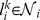 represents the pixel index of the 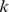
th neighbor within an image patch, 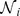, surrounding pixel 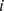, and 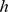 is a smoothing parameter.

### An NLM Filter for Dynamic PET Images

#### Noise in PET Images

PET sinogram data can be modeled as Poisson random vectors [40]. The Poisson mean is dependent on the true radiotracer distribution through the physical model of the system and may also depend on unwanted statistical effects such as scatter and random coincidences. Most iterative reconstruction approaches that retrieve the PET image (the true radiotracer distribution) from the sinogram data are nonlinear. Due to the nonlinearity, computation of the statistical properties of the reconstructed PET image directly from those of the data is a challenging problem. Sophisticated techniques to approximate the mean and covariance of reconstructed PET images have been the focus of a wide range of papers [41]–[43], which have been reviewed in [44]. These methods generally model the reconstructed image as a Gaussian random vector with mean and covariance that have a complex functional dependence on the forward model and the reconstruction parameters, including estimator-dependent parameters (e.g. the regularization parameter) and algorithm-dependent parameters (e.g. the iteration number). The Gaussian noise assumption is therefore widely prevalent in the context of dynamic PET [45], [46]. Accordingly, in this work, we assume that the reconstructed PET images are corrupted by Gaussian noise with unknown mean and variance. Additionally, for a frame-by-frame dynamic PET acquisition, the time bins for the earlier parts of the scan are kept short to capture the fast kinetics right after tracer injection. As a result, the earlier time frames in a typical dynamic PET image have fewer photon counts and are more heavily corrupted by noise than the later time frames.

#### Spatiotemporal Patches

One of the underlying ideas of this work is that image-based similarity information derived from the less noisy later time frames of a dynamic PET image can be used to effectively denoise the entire time series, including the noisier earlier time frames. The NLM framework has been shown to allow robust image-based similarity computation using image patches. To tap the full potential of the NLM filter for denoising dynamic PET images, we exploit the additional temporal dimension in these images by using spatiotemporal patches for similarity computation. As in the original NLM formulation, the immediate local neighborhood determines the spatial composition of the patch. In determining the temporal component of each patch, we impose a temporal threshold, 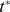, such that the temporal component is limited to only (less noisy) later time points 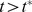 in the time series. We denote intensity values in the noisy reconstructed dynamic PET image as 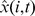, where 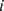 represents the voxel index (spatial location) and 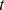 represents the time point. The weights are computed as follows:
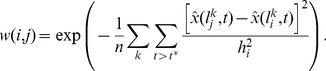
(3)


**Figure 1:**
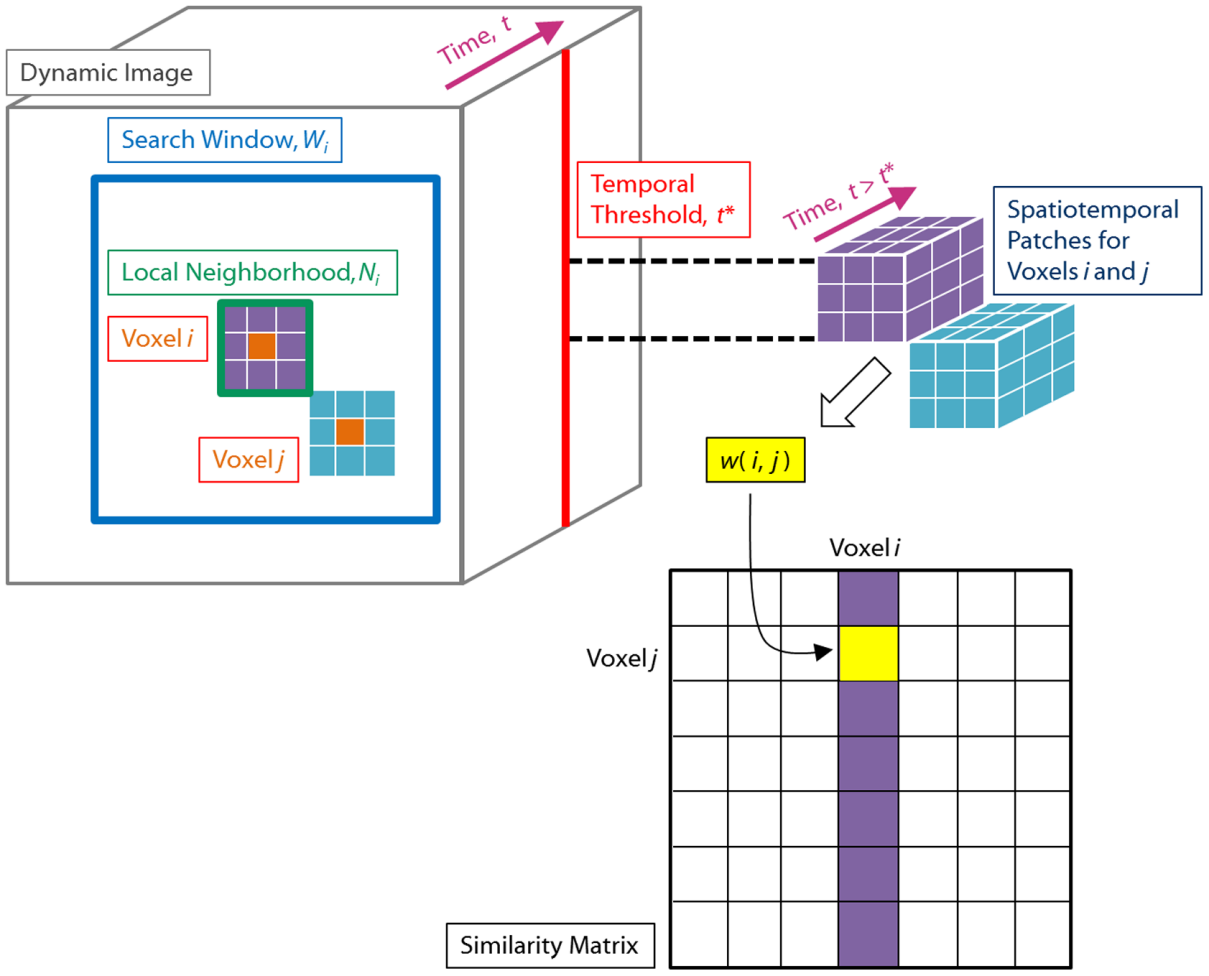
Similarity computation. The similarity between a voxel 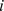 and a voxel 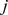, given by (3), is derived from spatiotemporal patches at the two voxels, composed of the local spatial neighborhood (denoted 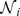 for voxel 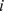) and all time points beyond a temporal threshold 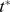. The pairwise weights can be assembled into a symmetric matrix of similarities as shown.

Here 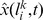 is the intensity of the 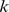
th neighbor of voxel 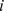 at time 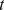, 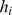 is the smoothing parameter for voxel 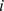, and 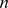 is the total number of elements in each spatiotemporal patch (e.g. 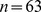 for a 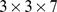 spatiotemporal patch spanning a 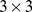 square in image space and 7 time frames). Since the variance of the reconstructed PET image is spatially variant, we have introduced a spatially varying smoothing parameter 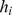. The introduction of 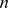 in (3) makes the choice of 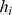 less sensitive to patch size variation. Figure 1 illustrates this process flow leading to the computation of a matrix of non-local similarities. The denoised TACs can now be computed from these similarities as follows:
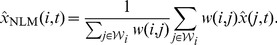
(4)


#### Parameter Adjustment

The proposed NLM denoising framework has four key parameters that require tuning: the size of the local spatial neighborhood, 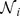, the temporal threshold, 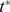, the size of the search window, 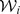, and the smoothing parameter, 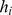. The selection procedures for these parameters are discussed below:

- *Local Neighborhood,* 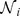: For most image restoration applications, the patch size is set to 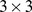 or 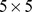 for color images and 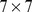 or 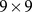 for grayscale images [15]. Since our patches contain additional information from the temporal component, the local neighborhood is set to 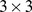 in the interest of computational speed.
- *Temporal Threshold,* 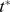: Dynamic frame-by-frame acquisition protocols for 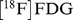 commonly use longer time bins after the first 30 min of acquisition. Accordingly, we set 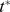 to 20 min for the simulation and experimental studies described in this paper.
- *Search Window,* 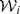: While the imposition of a spatially restricted search window in place of the entire image is in conflict with the overall non-local philosophy, it has been shown that the use of a large search window may add significant bias to the denoised image [24]. This is because the exponential form of the similarity metric generates small but non-zero contributions from very dissimilar patches. For a very large search window, the total contribution from dissimilar patches may turn out to be substantial in some cases. Remedies include use of a small search window [15], [25], use of adaptive search windows [47], or replacement of the exponential weights by functions with compact support [18], [39], [48]. We ran some preliminary simulations involving varying window sizes (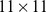, 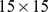, and 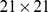) and found that the performance of the filter is relatively insensitive to the window size. Given our small local neighborhood size of 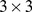, this is in agreement with Duval et al., which remarks that the impact of the window size tends to be larger for larger patch sizes. We, therefore, set the search window to a fixed size of 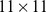 in the interest of computational speed.
- *Smoothing Parameter,* 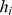: A number of approaches have been proposed for choosing the smoothing parameter for NLM. Optimal parameter selection using Stein's unbiased risk estimate has been shown to be particularly effective [20], [24]. In many applications involving additive or multiplicative white noise, the smoothing parameter is set to a constant multiple of the noise standard deviation [15], [19]. Kervrann et al. interpret the NLM filter with 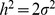 as a minimum mean square error (MMSE) estimator for an additive white Gaussian noise model with a noise variance of 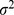 [21]. Computing accurate voxel-wise noise variance estimates for reconstructed PET images is a mathematically challenging and computationally intensive task [49]. Instead, we use the variance, 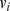, within the spatiotemporal patch for a voxel as a working approximation for the local variance. For a 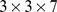 spatiotemporal patch, for example, the variance is computed across the 63 patch elements. For later time points, the PET tracer uptake is usually slowly varying. In fact, in regions associated with a negligible kinetic microparameter 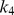, the system can be assumed to reach a pseudo-steady state at these later time points [8], [9]. Since we are working with a very localized spatial neighborhood of only 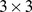 voxels and since the temporal component is slowly varying, the variance in the spatiotemporal patches is largely attributable to the noise variance. The smoothing parameter is then set to 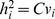, where 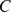 is a global constant, typically in the range 0.5 to 2. As mentioned before, the introduction of 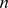 in (3) makes the choice of 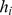 less sensitive to patch size variation.

## Methods

### A Simulation Study

#### Dynamic Digimouse Phantom

In order to generate a realistic simulation environment for testing the NLM denoising technique for dynamic PET images, we constructed a dynamic digital mouse phantom. Starting with the Digimouse atlas (http://neuroimage.usc.edu/Digimouse.html) [50], [51], a labeled atlas based on co-registered CT and cryosection images of a 28g nude male mouse, we identified regions representing the following tissue types: muscle, brain (minus the cerebral cortex), cerebral cortex, heart, bladder, stomach, spleen, pancreas, liver, kidney, and lung. We introduced two additional tissue types – skin and hepatic lesions – not present in the Digimouse atlas.

**Figure 2:**
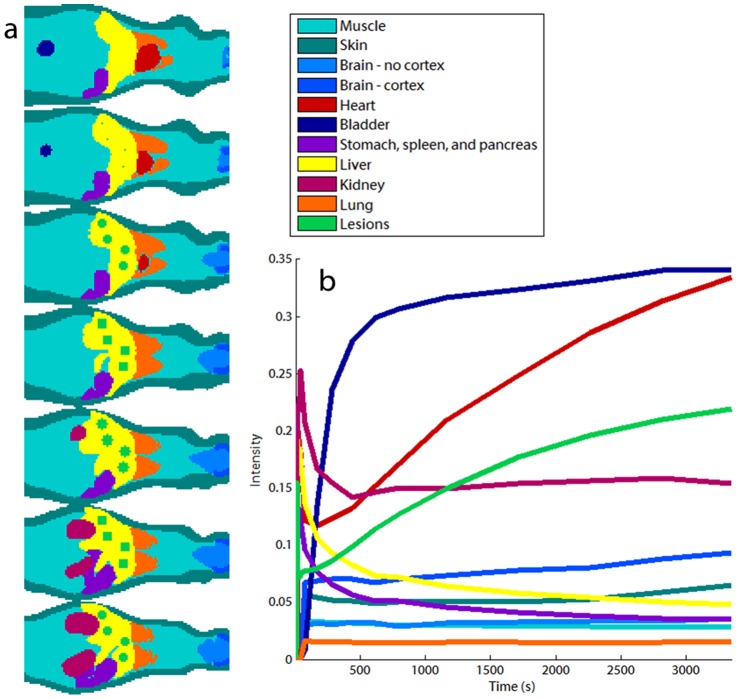
Dynamic digital mouse phantom. (a) Coronal slices of the Digimouse atlas showing the spatial distribution of distinct tissue types used in simulation. (b) Decay-corrected and averaged TACs corresponding to each manually delineated tissue type extracted from a dynamic PET image of a mouse. The color codes for different tissue types used for both the spatial map and the TACs are indicated in the legend.

The TACs for all the distinct regions (except for the lesions) were extracted from a real preclinical dynamic 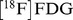 PET dataset acquired for a mouse using a microPET Focus 220 small animal imaging scanner. All procedures were performed with approval from the University of Southern California Institutional Animal Care and Use Committee (IUCAC) obtained satisfying appropriate protocol requirements. The plasma input function was obtained by manual sampling from the femoral artery of the animal. Dynamic frame-by-frame PET images were reconstructed using 2 iterations of the 3D ordered subsets expectation maximization (OSEM) algorithm with 12 subsets followed by 18 iterations of the maximum *a posteriori* (MAP) algorithm [52], [53]. Uniform ROIs for each tissue type were delineated by hand, and the decay-corrected TACs within each ROI were averaged to obtain reliable denoised estimates of tracer kinetics. The TACs for the lesions were generated using a three-parameter compartment model using published values of tumoral kinetic parameters: 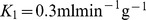, 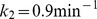, 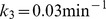, and blood volume fraction =  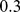 [54]. The spatial distribution of the tissue types and the corresponding TACs are showing in figure 2. For reference, the kinetic parameters obtained by fitting a three-parameter compartment model to the organ-wise TACs in figure 2(b) are provided in table 1. The size of the dynamic phantom was 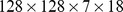 with three spatial dimensions, one temporal dimension, and 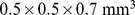 voxels. The simulated lesions were spherical in the image space with a diameter of 6 voxels.

**Table 1:** Fitted kinetic parameters for the organ-wise TACs displayed in figure 2(b).

Tissue type	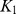 (ml min 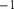 g 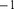 )	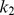 (min 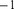 )	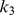 (min 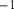 )
Muscle	0.1128	0.3166	0.0167
Skin	0.3145	0.6513	0.0320
Brain - no cortex	0.1063	0.3114	0.0253
Brain - cortex	0.2361	0.3239	0.0339
Heart	0.9500	0.9114	0.1024
Bladder	0.4343	0.0935	0.0178
Stomach, spleen, and pancreas	0.2501	0.3443	0.0032
Liver	0.2532	0.2382	0.0038
Kidney	0.6500	0.3785	0.0207
Lung	0.0559	0.3426	0.0216
Lesions	0.3000	0.9000	0.0300

#### Noisy Data Generation

In order to generate noisy dynamic images, the frame-by-frame static images from the dynamic Digimouse phantom were first forward projected to create noiseless sinograms. Noisy data was generated using Poisson deviates of the projected sinograms, a noise model widely accepted in the PET imaging community [55], [56]. The Poisson deviates were generated with a mean of 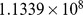 counts for the full scan duration of 3593.5 s. The mean count for each individual sinogram was determined by scaling the mean count for the full duration by a factor dependent on the activity per frame and the frame duration. The frame durations used in simulation corresponding to 18 temporal bins (from the preclinical acquisition described above) are as follows: 0.5 s, 2 s, 4 s, 6 s, 3 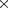 10 s, 60 s, 2 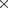 120 s, 3 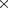 180 s, 4 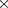 550 s, and 511 s. The noisy sinograms were then reconstructed frame by frame using 15 OSEM iterations with 21 subsets.

#### Reference Denoising Techniques

In order to assess the performance of NLM relative to other denoising techniques for dynamic PET, we compare the denoising capability of this method with four other denoising approaches:

- *Gaussian denoising*: The first reference method used is the traditional Gaussian denoising approach, where a 2D Gaussian-weighted kernel is used to compute a local, spatial average at a given voxel. Gaussian denoising is chosen as a reference, in part, because of its universality. In addition, we treat it as representative of the important class of denoising filters that rely solely on local averaging.
- *PCA based denoising*: As a second reference, we use principal component analysis (PCA) based denoising. This method applies singular value decomposition along the temporal dimension and generates a low-rank spatiotemporal approximation of the dynamic image. We picked this method for comparison because of its popularity in the specific context of handling time series data [57], [58]. In addition, we treat it as a representative of the class of denoising methods for time series datasets that rely on a set of temporal basis functions. PCA is based on the Karhunen Loève transform, which generates an orthogonal temporal basis set that best explains the variance in the time series data. Each TAC is represented as a linear combination of this temporal basis set. Assuming that the variance in the principal components corresponding to the smaller eigenvalues is chiefly due to noise, these components can be suppressed to obtain a denoised time series vector.
- *HYPR denoising*: The third reference method used is HighlY constrained backPRojection (HYPR), which has been applied to denoise both dynamic MRI [59] and dynamic PET [60] images. In this technique, a time-averaged composite image is first generated from the spatiotemporal image. Next a box-filtered version of the spatiotemporal image is divided by a box-filtered version of the composite image to create a weighting image. The HYPR denoised image is obtained by multiplying the composite image by this weighting image. It has been shown that this technique improves the signal-to-noise ratio (SNR) of the individual frame-by-frame images by utilizing the high SNR of the composite image.
- *Conventional NLM denoising*: As a fourth reference approach, we use the conventional NLM filter based on (1) and (2). The filter is applied to the spatiotemporal images slice by slice and time frame by time frame. The similarity metric is based on 2D spatial patches. A uniform smoothing parameter is used for all slices and time points. From this point on, we refer to the convention NLM technique (based on spatial patches) as NLM-S and the proposed technique (based on spatiotemporal patches) as NLM-ST.

#### Patlak Parametric Imaging

The targeted application in this work is the Patlak graphical technique [8], [9] for macroparameter fitting. This approach is only valid for irreversible binding of tracer in the second compartment, an assumption that is true for 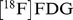 in most tissue types. This corresponds to the assumption that the microparameter 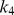 is negligibly small. The observed PET signal, 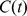, is dependent on tracer concentration in blood plasma, 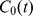. The Patlak technique plots the quantity 
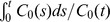
 (referred to as the stretched time or normalized time) against 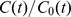 (referred to as the apparent volume of distribution). At later time points (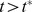), when the system can be assumed to have reached a pseudo-steady state, this relationship is approximately linear:
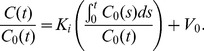
(5)


The Patlak method seeks to compute the slope 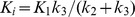 in (5), known as the Patlak influx constant, and the intercept 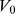, referred to as the initial volume of distribution [6]. The Patlak influx constant, which represents the net tracer uptake at steady state into the second compartment where irreversible binding takes place, is the macroparameter that will be examined in the simulation and experimental studies presented here.

#### Evaluation Metrics

The mean squared error of an estimator can be decomposed as a sum of the squared bias and the variance [61]. For any imaging inverse problem involving a tunable smoothing parameter, as the degree of smoothing is increased, the estimator variance decreases while the estimator bias increases and vice versa. We rely on this bias-variance trade-off as the figure of merit for comparative assessment of different denoising methods in our simulations. We plot absolute bias vs. standard deviation curves for each ROI and for the overall volume using [62]:
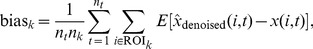
(6)

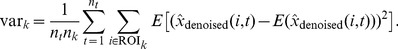
(7)


Here 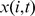 and 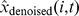 are the true and denoised spatiotemporal images respectively, 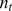 represents the number of discrete time frames, 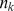 represents the number of voxels in 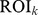, the 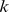
th ROI, and the expectation operation 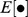 is performed over multiple noise realizations.

To assess the impact of different denoising methods on parametric images in our simulation study, we also plot bias vs. standard deviation curves for the Patlak parametric images computed from the denoised dynamic images. The equations for bias and variance for this case are analogous to (6) and (7) except for the averaging operation over the temporal dimension.

### Experimental Studies

#### Preclinical Study

A preclinical dynamic 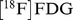 PET scan was performed on a mouse using a microPET Focus 220 scanner for dedicated high-resolution small animal imaging. The 27 g mouse was kept in a fasting state for 4.5 hours and anesthetized with 2% isoflurane. The injected 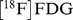 dose was 8.4952 MBq with an injection volume of 60 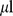. The plasma input function was obtained by manual sampling from the femoral artery of the animal. Data was acquired frame by frame for 60 min. The frame durations corresponding to 18 sampling time points for the acquisition were as follows: 0.5 s, 2 s, 4 s, 6 s, 3 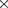 10 s, 60 s, 2 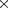 120 s, 3 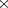 180 s, 4 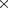 550 s, and 511 s. All procedures were performed with approval from the USC IUCAC obtained satisfying appropriate protocol requirements. Dynamic PET images were reconstructed using 2 iterations of the 3D OSEM algorithm with 12 subsets followed by 18 iterations of MAP with a uniform quadratic penalty and a regularization parameter of 0.1.

#### Clinical Study

Clinical data was acquired from a patient with hepatocellular carcinoma immediately after injection of 375 MBq of 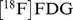 using a Siemens Biograph system. The plasma input function was image-derived and determined using an averaging operation on undenoised TACs within a manually delineated aortic region. The same input function was used for computing parametric images for different denoising methods. Data was acquired in list mode for 60 min and later binned to 30 frames. The frame durations corresponding to 30 sampling time points for the acquisition were as follows: 8 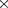 15 s, 4 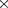 30 s, 11 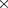 60 s, 5 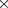 300 s, 2 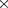 600 s. All procedures were performed with written informed consent and with approval from the University of Southern California Institutional Review Board obtained satisfying appropriate protocol requirements. Reconstruction was performed using 2 iterations of 3D OSEM with 21 subsets followed by 18 iterations of MAP with a uniform quadratic penalty and a regularization parameter of 0.1.

#### Evaluation Metrics

For the experimental datasets, it is not possible to perform bias-variance analysis since the ground truth is unknown. For these datasets, we compute Patlak parametric images and examine two image quality metrics. The use of Patlak analysis for identifying tumor burden has been demonstrated both in the context of diagnosis (e.g. for differentiating cancerous tumors from benign ones) and treatment monitoring (e.g. differentiating viable cancerous tissue from surrounding scar tissue after radiotherapy or chemotherapy) [63]–[65]. We therefore compare the different denoising approaches in terms of their ability to preserve high intensity features in the Patlak images. Accordingly, the first metric examined is the percentage recovered signal in a hot ROI (the signal region) in the denoised Patlak images relative to the undenoised Patlak image computed directly from the noisy dynamic images. However, this metric by itself fails to capture the noise characteristics of the generated images. We therefore examine a second, more holistic metric – the contrast-to-noise ratio (CNR) [66], [67]. In clinical applications such as treatment monitoring, where the goal is to detect potentially low remnant activity after therapy, the CNR of the Patlak images can be quite critical. To compute the CNR, in addition to the signal region, we identify a low intensity area expected to have relatively uniform uptake (the background region). The CNR is then computed by dividing the difference between the mean intensities of the signal and background regions by the standard deviation in background region.

## Results

### Simulation Results

#### Bias-Variance Analysis

**Figure 3:**
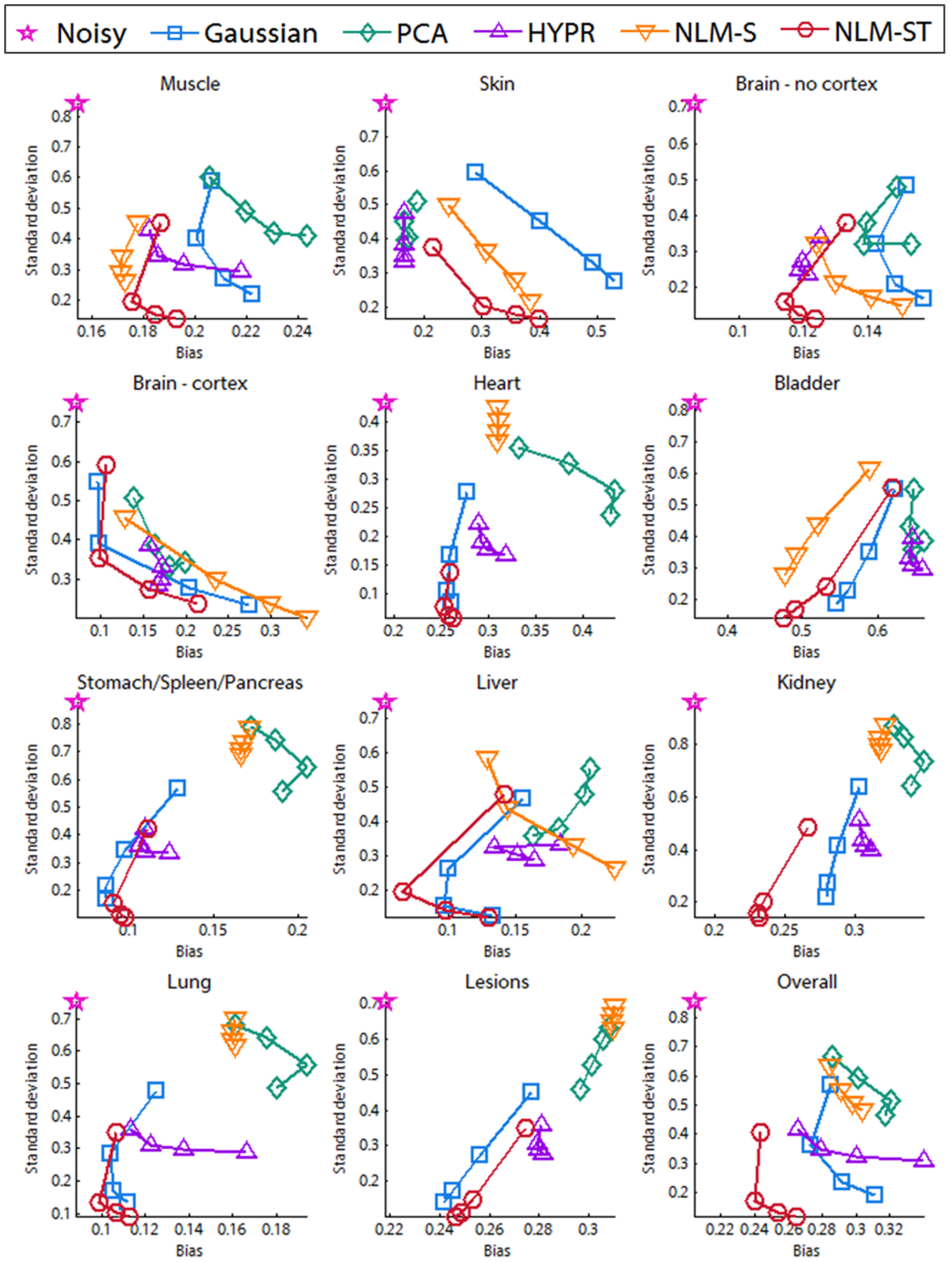
Plots of bias vs. standard deviation. Percentage bias vs. percentage standard deviation plots are shown for the 11 ROIs (indicated in figure 2) and for the overall phantom volume for the noisy and denoised dynamic images. Of the five denoising methods compared (Gaussian, PCA, HYPR, NLM-S, and NLM-ST), NLM-ST simultaneously yields lowest bias and lowest standard deviation for a majority of the individual ROIs and also for the overall volume.

Using the setup described for generating noisy dynamic images, we created 20 noisy realizations of the dynamic phantom image. For Gaussian filtering, the kernel size is fixed to 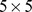 pixels while the standard deviation is swept over the following range of values: 0.5, 0.7, 1.0, and 2.0, which correspond to full widths at half maximum (FWHM) of 0.59 mm, 0.82 mm, 1.18 mm, and 2.35 mm respectively. For PCA, the number of principal components used to generate a low-rank approximation of data was varied over the range of values: 8, 6, 4, and 3. For HYPR, the box filter size was set to 3, 5, 7, and 11. For NLM-S, the smoothing parameter was set at 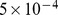, 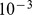, 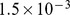, and 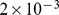. For NLM-ST, the smoothing parameter was set using 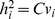 with the global parameter, 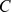, set to the following values: 0.5, 1.0, 1.5, and 2.0. Percentage absolute bias vs. percentage standard deviation curves were computed for each of the 11 different ROIs as well as for the overall volume as shown in figure 3. The plot of overall bias vs. standard deviation indicates marked decrease in both bias and standard deviation achieved using NLM-ST relative to the other techniques. When compared to Gaussian denoising, the improvement achieved using NLM appears most substantial in ROIs with the most numerous voxels since, for these ROIs, a multitude of similar voxels can be found within the search window. NLM-ST also substantially outperforms Gaussian denoising in the lesions. This can be attributed to the localized nature of this ROI, due to which spill-in from the surrounding tissue caused by the Gaussian filter leads to a significant biasing effect. PCA denoising seems to generate images with relatively high variance compared to the other methods. The PCA-denoised image retains a set of the slower varying principal components (a subset of the temporal basis that accounts for as much of the variability in the data as possible). While very effective for time series datasets that can be described using a smaller set of basis functions, PCA seems less effective for whole body applications which may involve a large range of complex TACs. As more principal components are suppressed, the variance decreases. But, since only a few basis functions are used to describe the entire dataset, PCA seems to favor a few ROIs (as seen from the low bias in the skin and the lungs), while potentially generating bias and image artifacts in some of the other ROIs. The HYPR technique generates higher variance in most regions compared to NLM-ST. Like PCA, it generates very low bias in the skin region. Increase of the box filter dimensions leads to significant spillover, which is particularly noticeable in the lesions in the last time frame. The NLM-S technique generates low bias in the muscle tissue. For most localized ROIs, such as the lesions, the kidneys, and the heart, this method leads to both high bias and high variance. The poor performance is partly attributable to the susceptibility of the similarity metric in the early time frames to the high noise levels and also partly due to the ineffectiveness of a uniform smoothing parameter for this application.

**Figure 4:**
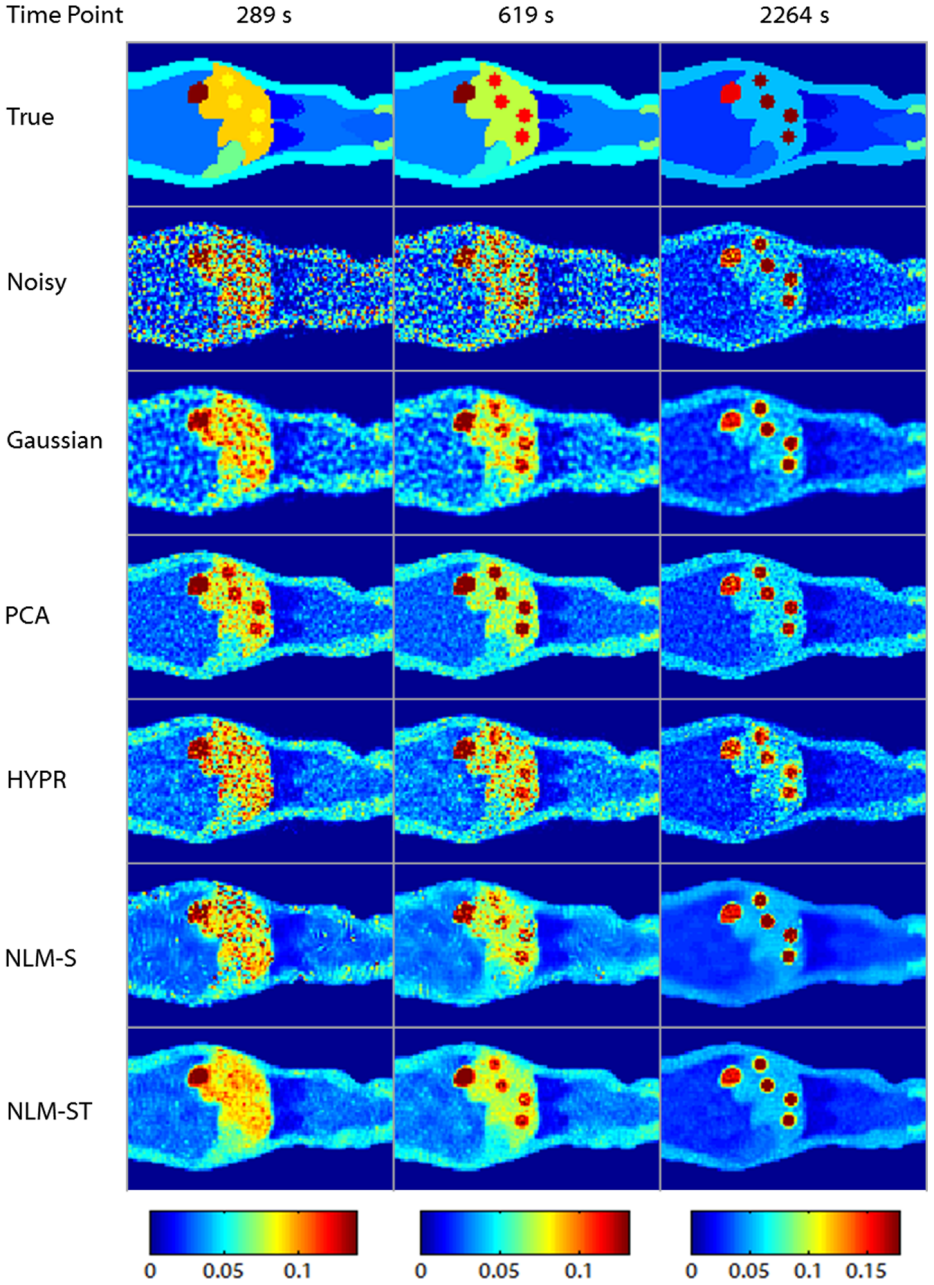
A coronal slice from the dynamic Digimouse phantom. The rows represent the true, noisy, Gaussian-denoised, PCA-denoised, HYPR-denoised, NLM-S denoised, and NLM-ST denoised images respectively. The columns represent three time points (289 s, 619 s, and 2264 s) reflecting the evolution of activity over time. The columns correspond to time bin sizes of 120 s, 160 s, and 550 s from left to right. Accordingly, the left and middle columns are noisier than the right column.

Coronal slices from the original, noisy, and denoised spatiotemporal images are displayed in figure 4. The tuning parameter choices for the displayed denoised images were based on the optimal overall bias-variance performance for each method as revealed in figure 3. The Gaussian denoised image was obtained using a Gaussian filtering kernel with a standard deviation of 0.7 (0.82 mm FWHM). The PCA denoised image was based on 6 principal components. The HYPR denoised image was based on a box filter size of 5 voxels. The NLM-S denoised image was generated using a smoothing parameter of 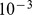. The NLM-ST denoised image was generated using a global smoothing constant 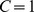. The columns represent three time points (289 s, 619 s, and 2264 s) reflecting the evolution of activity over time. From left to right, the columns correspond to time bin sizes of 120 s, 180 s, and 550 s. Accordingly, the left and middle columns are more corrupted by noise than the right column. It should be noted that the image quality improvement in the earlier frames achieved using NLM-ST is quite significant compared to all the other methods. Compared to NLM-ST, the NLM-S 2264 s time point image is oversmoothed (blurred boundaries between the lungs and the muscle), while the earlier time point images are clearly undersmoothed. When we focus on more minute high-intensity features such as the lesions, we observe that they are noisier and less structured with Gaussian, HYPR, and NLM-S denoising at 619 s (middle column) and have significant artifacts with PCA denoising, prominently visible when we look at the 289 s time point (left column). For both NLM-S and NLM-ST, we observe some artifacts appearing as remnant structured noise. These are most prominent in the low count muscle region. Due to the small spatial extent (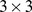) of the patches used in this work, the NLM technique tends to reinforce some noise patterns that it misinterprets as texture. These artifacts can be suppressed by resorting to patches with a larger spatial extent.

#### Patlak Analysis

**Figure 5:**
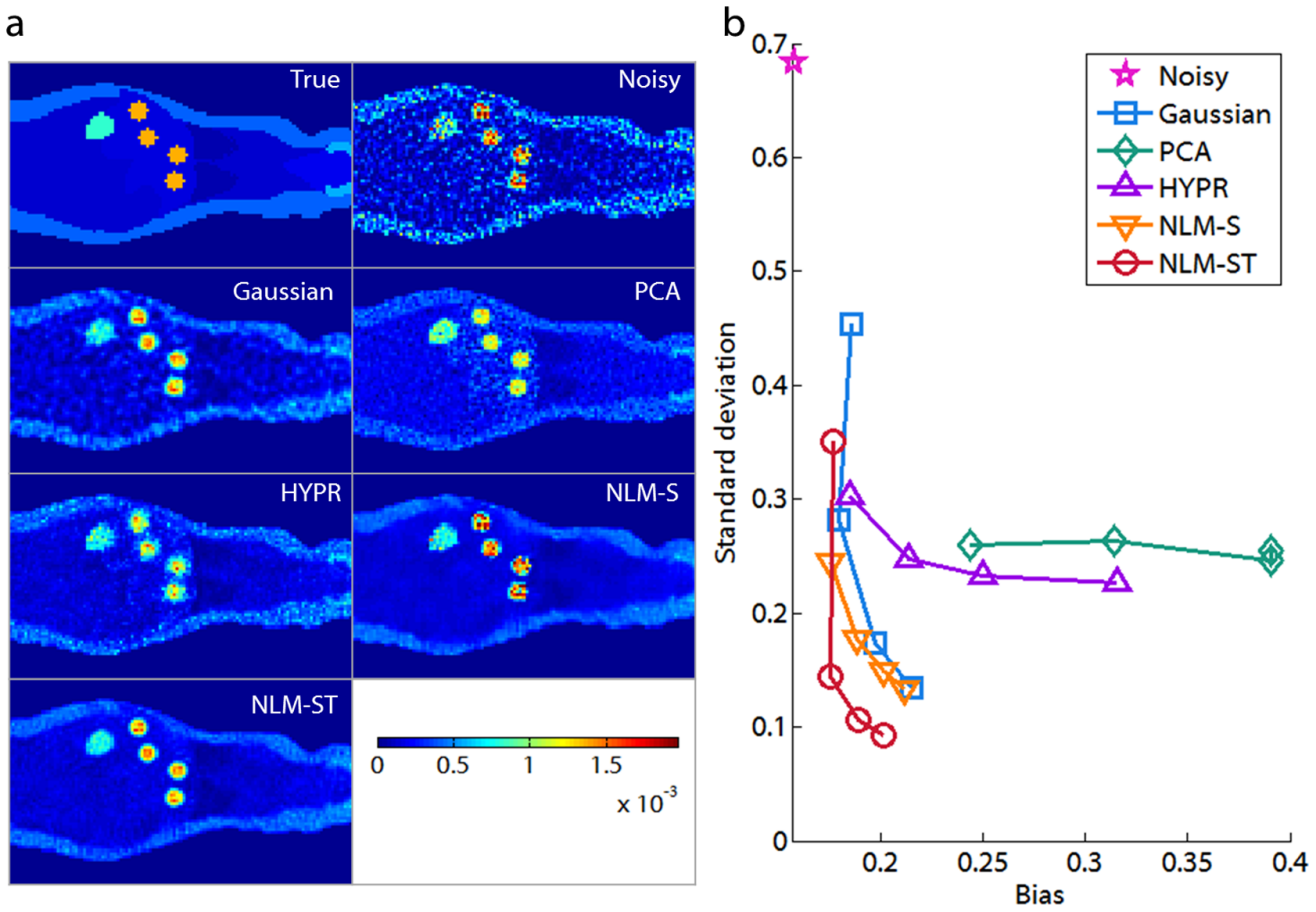
Patlak parametric imaging for the digital phantom study. (a) The Patlak influx constant 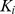 was computed from the true, noisy, Gaussian-denoised, PCA-denoised, HYPR-denoised, NLM-S denoised, and NLM-ST denoised images of the dynamic Digimouse phantom. (b) Plots of overall percentage bias vs. percentage standard deviation for the Patlak parametric images computed from the noisy and denoised dynamic images.

To explore the utility of NLM-ST denoising in the model-based interpretation of dynamic PET images, we use the Patlak graphical technique for macroparameter fitting. Figure 5(a) shows a coronal slice from the volumetric Patlak parametric maps corresponding to the true, noisy, Gaussian-denoised, PCA-denoised, HYPR-denoised, NLM-S denoised, and NLM-ST denoised images displayed in figure 4. Figure 5(b), which shows plots of the overall bias and variance in the Patlak parametric maps corresponding to the five denoising techniques, demonstrates that the simultaneous low bias and low variance behavior observed in the NLM-ST denoised spatiotemporal images also reflect in the corresponding macroparametric estimates, thereby offering improved quantitation. It should be noted that, while the NLM-S denoised dynamic images exhibited high overall bias and variance, for Patlak parametric imaging, the performance of this method is better than all the other methods except for NLM-ST. This apparent performance improvement is due to the fact that the Patlak macroparameter estimate does not depend on earlier time points, where NLM-S has particularly poor bias-variance performance due to the uncertainty in the similarities computed from these noisier time frames.

## Experimental Results

### Preclinical Data Analysis

**Figure 6:**
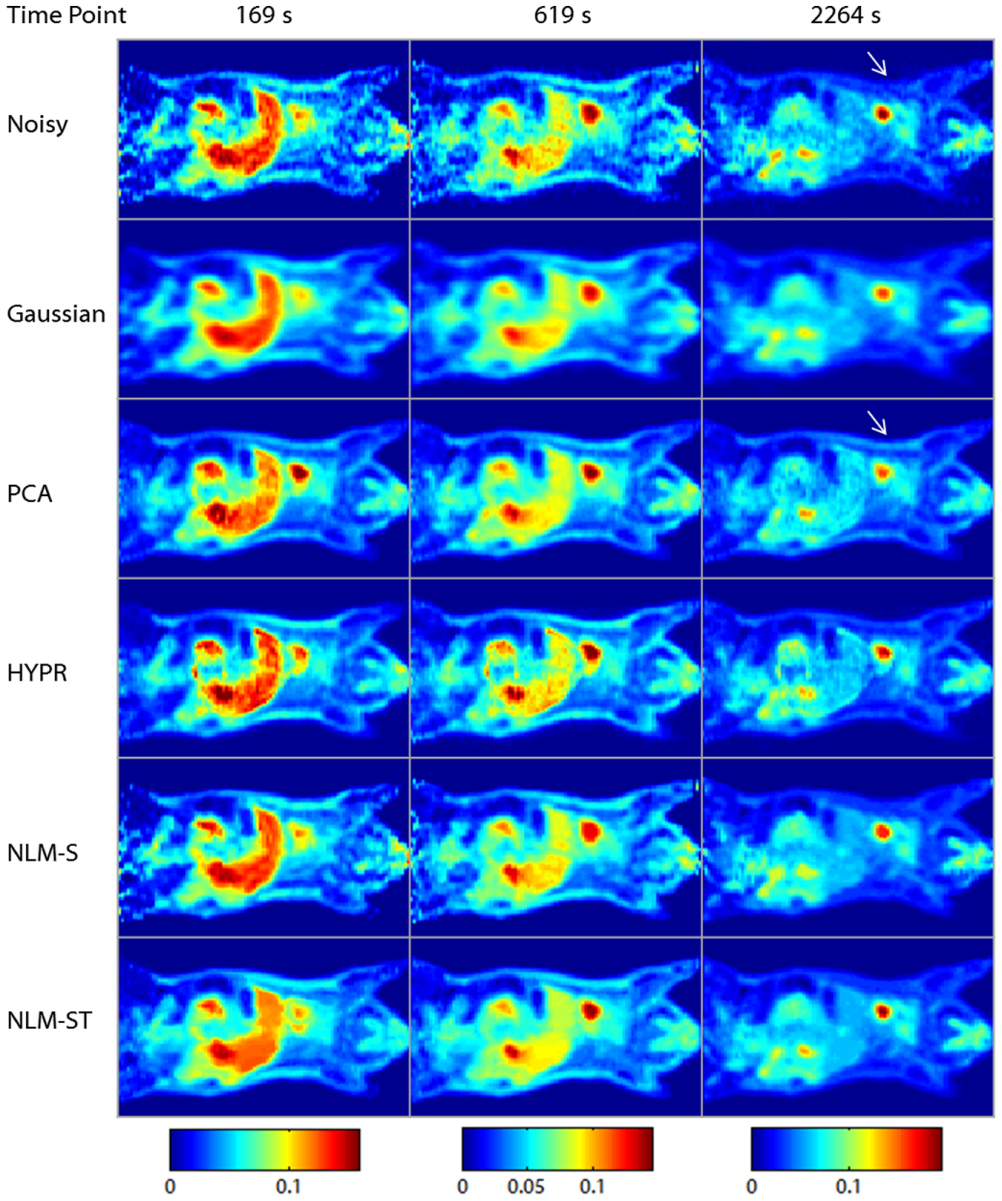
A coronal slice from the dynamic 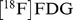 PET image of a mouse. The rows represent the noisy, Gaussian-denoised, PCA-denoised, HYPR-denoised, NLM-S denoised, and NLM-ST denoised images respectively. The columns represent three time points (169 s, 619 s, and 2264 s from left to right) reflecting the evolution of activity over time. The white arrows pinpoint extra uptake in the skin in the later frames of the PCA-denoised image, which appears to be an image artifact.

Coronal slices from the noisy and denoised spatiotemporal preclinical images are displayed in figure 6. The NLM-ST filter was based on spatiotemporal patches of size 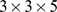 (two spatial dimensions and one temporal dimension), a search window of size 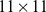, and 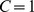. The Gaussian filter used had a kernel diameter of 7 voxels and a standard deviation of 2 (1.88 mm FWHM). The PCA approach uses the 5 largest principal components chosen based on the singular value spectrum. Suppression of fewer principal components leads to significant artifacts in some regions, while inclusion of more components seems to substantially increase the variance in the background muscle tissue. The HYPR method was based on a box filter size of 7 voxels. The NLM-S smoothing parameter was set to 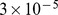. Visual comparison of the PCA image slice at 2264 s with the corresponding noisy image reveals that PCA generates some artifacts in the form of extra activity in the skin (indicated by white arrrows in figure 6). At the 2264 s time frame, relative to NLM-ST, most of the other methods generate poorer contrast in the mouse heart. However it should be noted that, while NLM-ST leads to a smoother image at the the earlier 169 s time point, it also leads to suppressed activity in the liver, which is likely to introduce some bias in the microparameters.

**Figure 7:**
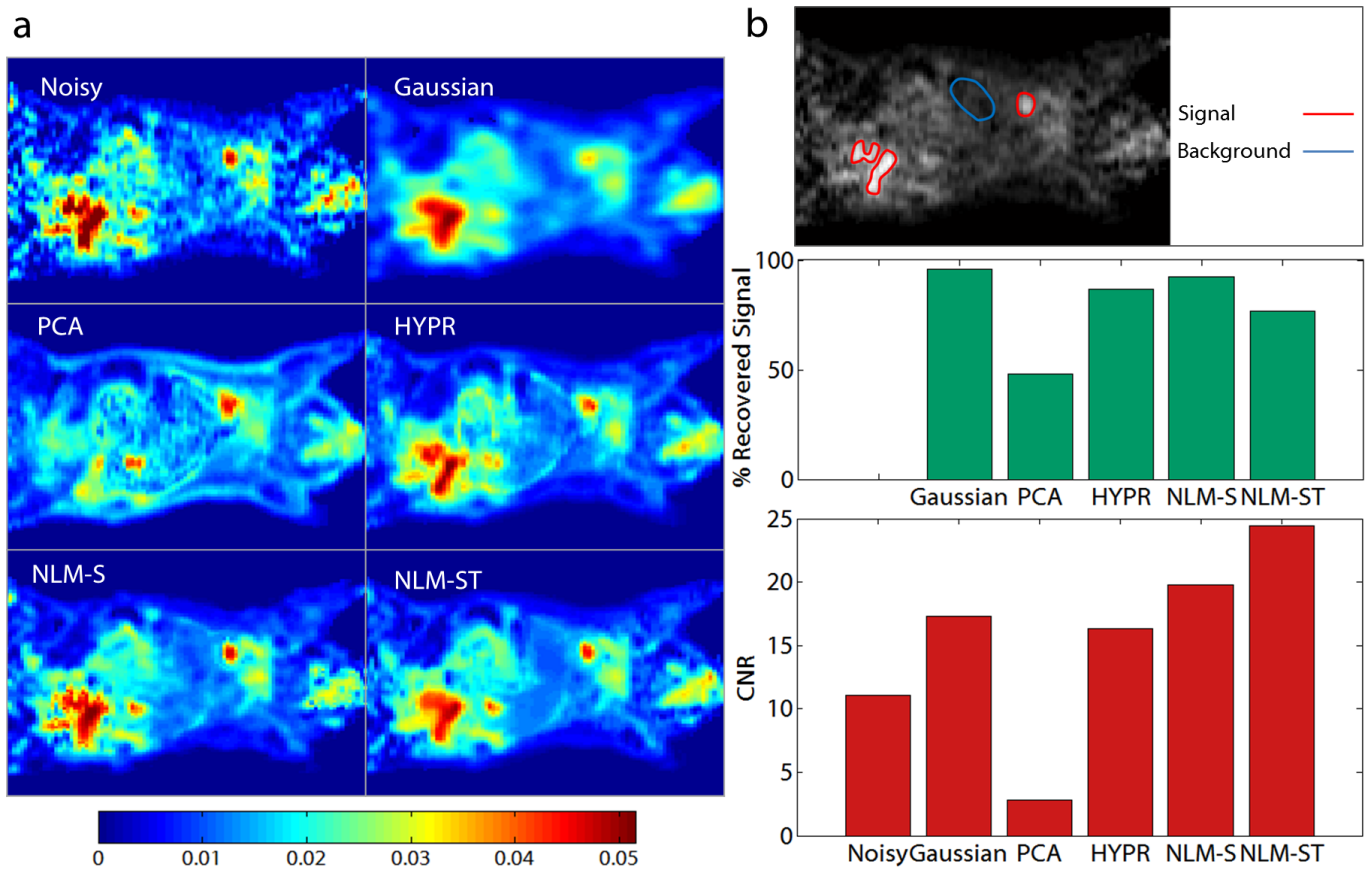
Patlak parametric imaging for the preclinical study. (a) The Patlak influx constant 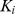 was computed using noisy, Gaussian-denoised, PCA-denoised, HYPR-denoised, NLM-S denoised, and NLM-ST denoised images from an 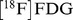 PET mouse study. (b) The top row delineates the signal (red) and background (blue) ROIs used for evaluation. The middle row shows the percentage recovered signal in the hot regions for different denoising methods. The bottom row shows the CNR in the Patlak parametric images, measured as the ratio of the contrast between the signal and the background ROIs to the standard deviation in the background.

For model-based interpretation of this dynamic dataset, we computed a spatial map of the Patlak influx constant. Figure 7(a) shows one coronal slice from the volumetric parametric image computed after denoising using Gaussian filtering, PCA, HYPR, NLM-S, and NLM-ST. We examined the percentage recovered signal in the heart and the hot regions in the lower abdomen for the different denoising methods relative to the noisy image. The high intensity signal region is delineated in the top row of figure 7(b). The percentage recovered signal displayed in the middle row of figure 7(b) was highest for Gaussian denoising, followed by NLM-S, HYPR, NLM-ST, and PCA respectively. To account for the noise behavior, we computed the CNR by dividing the difference between the mean intensities of the signal region and the background muscle tissue (a lower intensity region) by the standard deviation in the muscles (a region expected to have relatively uniform uptake). The signal and background ROIs used for computing the CNR are delineated in the top row of figure 7(b). The CNRs for the noisy and denoised Patlak images are displayed in the bottom row figure 7(b). The NLM-ST and NLM-S denoised images exhibit the highest CNR, with the former about 23.8% higher than the latter.

### Clinical Data Analysis

**Figure 8:**
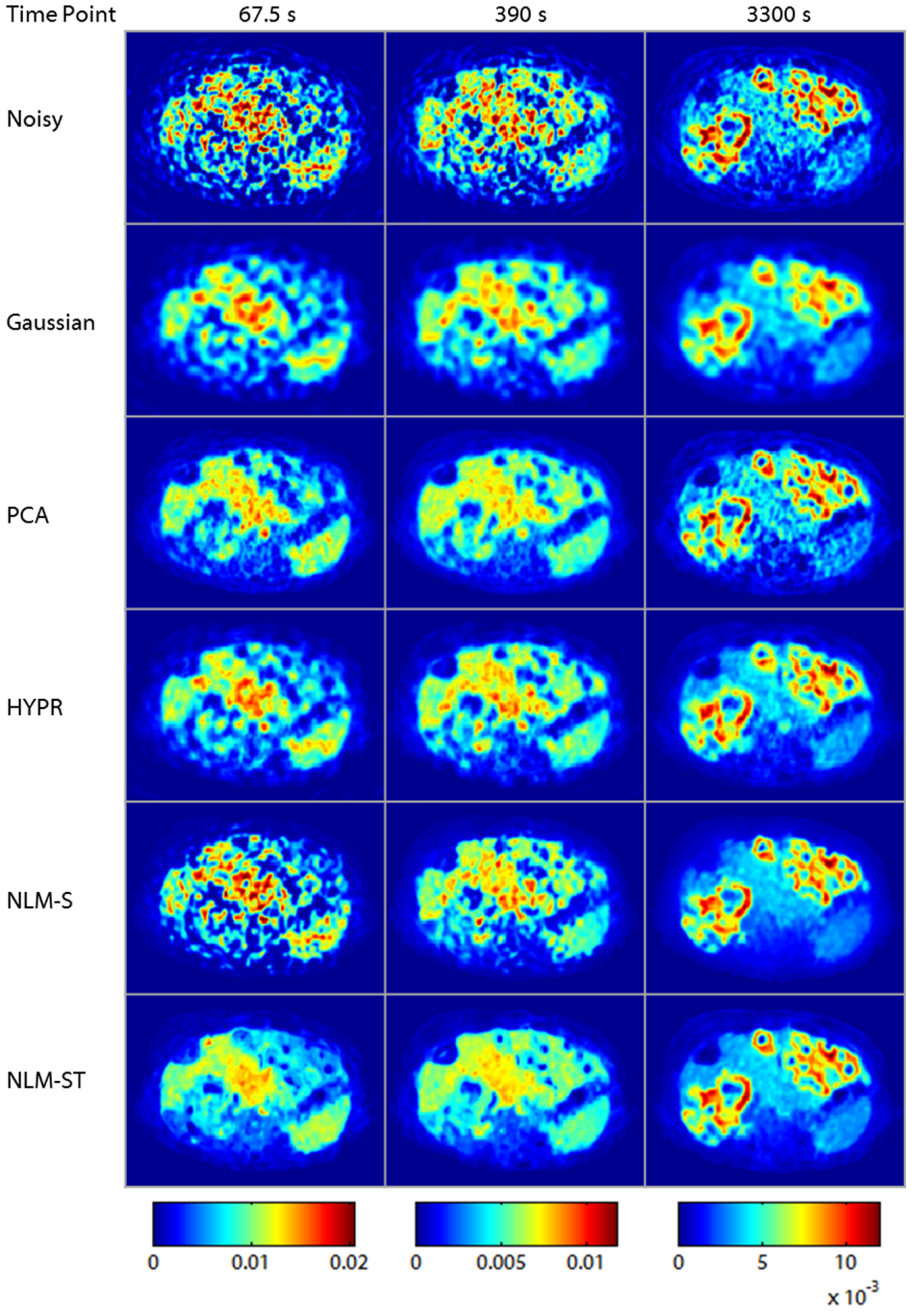
A transverse slice from the dynamic PET image of a patient with hepatocellular carcinoma. The rows represent the noisy, Gaussian-denoised, PCA-denoised, HYPR-denoised, NLM-S denoised, and NLM-ST denoised images respectively. The columns represent three time points (67.5 s, 390 s, and 3300 s from left to right) reflecting the evolution of activity over time.

**Figure 9:**
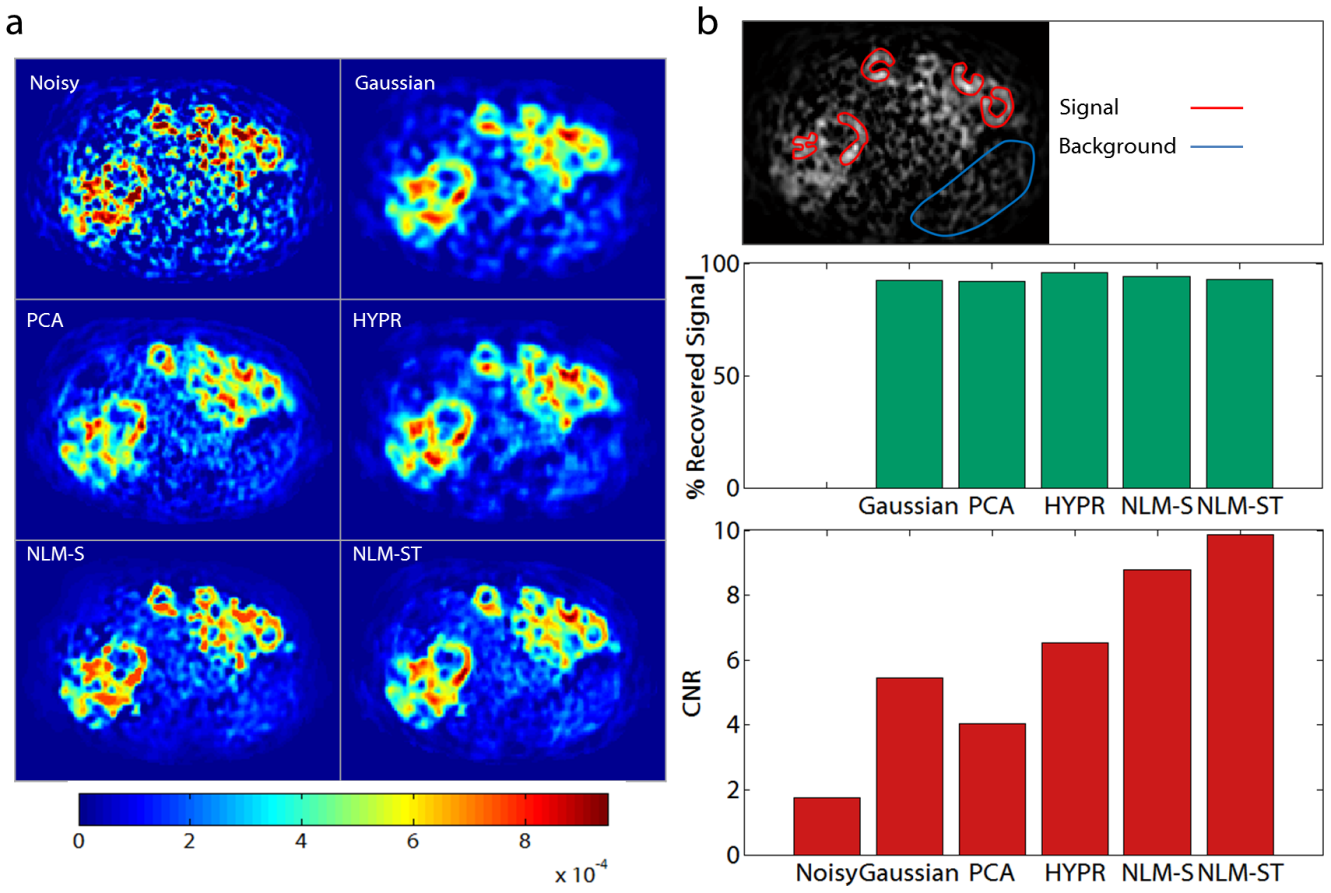
Patlak parametric imaging for the clinical study. (a) The Patlak influx constant 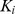 was computed using noisy, Gaussian-denoised, PCA-denoised, HYPR-denoised, NLM-S denoised, and NLM-ST denoised images from an 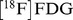 PET scan of a patient with hepatocellular carcinoma. (b) The top row delineates the signal (lesions marked in red) and background (spleen marked in blue) ROIs used for evaluation. The middle row shows the percentage recovered signal in the hot lesions for different denoising methods. The bottom row shows the CNR in the Patlak parametric images, measured as the ratio of the contrast between the signal and the background ROIs to the standard deviation in the background.

Transverse slices from the noisy and denoised spatiotemporal clinical images are shown in figure 8. The NLM-ST denoised image was generated using spatiotemporal patches of size 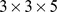, a search window of size 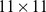, and 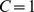. The Gaussian filter had a kernel size of 7 voxels and a standard deviation of 1.5 (7.15 mm FWHM). The PCA method employed the 10 largest principal components chosen based on the singular value spectrum. The HYPR method was based on a box filter size of 7 voxels. The NLM-S smoothing parameter was set to 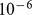. Visual comparison of the NLM-S and NLM-ST images reveals that, while the image quality for these methods during the later time frames appears comparable, the performance of the two methods are markedly different at 67.5 s (left column) and 390 s (middle column), where NLM-S leads to noisy and undersmoothed images. The late time frame behaviors of the different denoising methods are also manifested in the Patlak parametric maps shown in figure 9(a). The Patlak images highlight the uptake in the lesions inside the liver tissue. Evidently, this patient has multiple tumors in the liver. Several of the lesions are characterized by low-intensity necrotic cores and high intensity rings of viable tissue with high 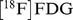 uptake [68], delineated in red in the top row of figure 9(b). The percentage recovered signal in the bright rings was comparable for all the methods examined as shown in the middle row of figure 9(b). The spleen, delineated in blue in the top row of figure 9(b), however, looks more uniform and distinct in the NLM-S and NLM-ST denoised Patlak images. Thus NLM-S and NLM-ST methods yield a generally smoother Patlak image with a more uniform non-tumorous background while preserving the lesion structure and the contrast between tumorous and non-tumorous tissue. These observations are confirmed by the CNR values in figure 9(b). The CNR for the liver Patlak images is computed by dividing the difference between the mean intensities of the lesions (a high intensity region) and the spleen (a lower intensity region) by the standard deviation in the spleen (a region expected to have relatively uniform uptake). The signal and background ROIs used for computing the CNR are indicated in the top row in figure 9(b). The NLM-ST and NLM-S denoised images exhibit the highest CNR, with the former about 12.2% higher than the latter.

## Discussion

We have adapted the NLM technique for the denoising of dynamic PET images. To test the performance of our method, we created a realistic dynamic digital mouse phantom based on the Digimouse atlas. We generated multiple noise realizations by reconstructing Poisson deviates of frame-by-frame sinograms. Using the simulated data, we compared the bias-variance characteristics of the developed spatiotemporal NLM technique (NLM-ST) with four other denoising approaches: Gaussian filtering, PCA, HYPR, and conventional NLM (NLM-S). Our results indicate that the NLM-ST technique has the lowest overall bias and variance and significantly outperforms the other methods. We then demonstrated that the low bias and low variance properties of the NLM-ST denoised dynamic images are also reflected in the Patlak macroparametric estimates, thus improving the accuracy of model-based quantitative interpretation. We applied our method to dynamic 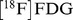 PET datasets from a mouse and from a liver cancer patient. Patlak parametric images for these datasets generated using the five denoising methods reveal clear and consistent improvement in CNR achieved using NLM-ST indicating that this method preserves image contrast for high intensity features while lowering the background noise variance. For one slice of the dynamic Digimouse atlas, the run times for the different denoising methods on a 3.33 GHz Intel  Xeon  X5680 system were: 0.05 s for Gaussian filtering, 1.2 s for PCA, 0.03s for HYPR, 16 s for NLM-S, and 2.36 s for NLM-ST. It should be noted that NLM-ST is faster than NLM-S because, unlike the latter which denoises one time frame at a time, the former uses spatiotemporal similarities to denoise the entire time series at one go. It should be noted that, in the NLM framework used in this work, the central patch was treated the same way as other patches. Also, since 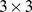 patches were used in the applications discussed in this work, no adhesion effect is visible in the restored images.

While NLM denoising has found wide applicability in MRI, to our knowledge, this is the first thorough investigation of its utility in dynamic PET, a particularly compelling application for denoising methods owing to the high noise levels in dynamic datasets. This paper introduces several key ideas that enable the employment of the basic NLM framework for denoising dynamic PET images. Firstly, this method exploits the less noisy image frames from later time points to denoise all time frames. Secondly, compared to our prior work involving temporal patches [39], this paper incorporates the concept of spatiotemporal patches derived from dynamic PET images. Finally, we introduce an automated approach to spatially vary the smoothing parameter for NLM. The majority of existing literature on NLM denoising deals with additive or multiplicative white noise and use a spatially invariant smoothing parameter. The noise levels in PET images are dose-dependent and vary substantially from one dataset to another, making parameter tuning critical for the overall success of the method. The NLM filter we described here computes a local variance estimate over each spatiotemporal patch, which accounts for the dependence of the noise statistics on the activity. This leaves us with a single global smoothing parameter which we set to a value in the range 0.5 to 2 for our simulation and experimental studies. Our simulation study indicated that all values in this range yield superior overall bias and variance properties compared to the reference methods.

The spatiotemporal patches used in this work are 3D (two spatial dimensions and one temporal dimension). In other words, the 4D spatiotemporal images were denoised slice by slice. A preliminary investigation on the efficacy of 4D spatiotemporal patches revealed a significant increase in computational burden without any major impact on image quality. Consequently, in the interest of computational speed, 3D patches were used in this work.

The spatial variation of the smoothing parameter is based on a local variance approximation over each spatiotemporal patch. Unlike applications involving images corrupted by white noise, PET exhibits a non-uniform voxel variance dependent on the activity, system model, reconstruction method, and other factors. The patch-based variance approximation is a simple and effective workaround. However, it tends to reduce the sharpness of edges in images, which is a limitation of this approach. The amount of local bias in the boundary voxels depends on the contrast between the uptakes in the organ of interest and the neighboring organ. In the last time frame of the NLM-ST denoised dynamic Digimouse PET images in figure 4, for example, the recovered activity in the neighborhood of the lesions is about 1.72 times the true value due to spillover from the lesions, slightly higher than the corresponding value of 1.65 for NLM-S. In the latter case, the spillover may be attributable to the rare patch effect [69]. Additionally, while the use of only the later time frames enables more robust estimation of non-local weights, it may have limitations in capturing the spatial structures that only appear in early time frames. This may lead to some spillover along boundaries due to potential oversmoothing in the earlier time point images. Due to the low counts (and hence high noise levels) in the early time frames, the uncertainty in the similarities computed directly from these frames is too large for these similarities to be reliable. Generally speaking, the benefits outweigh the risks. However, in these special cases, it is advisable to adjust the temporal threshold, if possible, to capture the contrast near the particular tissues of interest.

While a range of techniques exist for simultaneous reconstruction and denoising of all the dynamic frames using smoothing priors, a majority of these techniques only impose local smoothness. Tissue types exhibiting similar tracer dynamics are often distributed all over the body. Methods based on local smoothing priors fail to exploit this aspect. This is a key strength of NLM denoising. Also, for dynamic PET, spatiotemporal penalties enforcing smoothing along the temporal dimension or utilizing temporal basis functions have been used. But these tend to introduce temporal correlations in the images, which are undesirable for tracer kinetic analysis. In comparison, for our method, while the similarities are spatiotemporal, the averaging operation is purely spatial. An interesting topic to investigate in the future is the possibility of extending this formulation to design a non-local prior for dynamic image reconstruction. We will also investigate more sophisticated means for automatically tuning the smoothing parameter. Based on the success of NLM denoising for MR images, yet another interesting avenue to explore would be the derivation of self-similarities from both anatomical (MR) and functional (PET) images. The added MR contrast will enable more robust estimation of similarities in regions where the contrast in the later time frames of the PET is low. In addition, segmented anatomical images can also be used to guide our local variance estimation procedure, thus ensuring that only voxels belonging to the same tissue class are used for variance computation, thereby reducing spillover at boundaries.
